# Noncombustion Emissions of Organic Acids at a Site near Boise, Idaho

**DOI:** 10.1021/acsestair.4c00138

**Published:** 2024-11-27

**Authors:** Andrew J. Lindsay, Brigitte M. Weesner, Kyle Banecker, Lee V. Feinman, Russell W. Long, Matthew S. Landis, Ezra C. Wood

**Affiliations:** † Department of Chemistry, Drexel University, Philadelphia, Pennsylvania 19104, United States; ‡ US EPA, Office of Research and Development, Research Triangle Park, North Carolina 27711, United States

**Keywords:** Organic Acids, Chemical Ionization Mass Spectrometry, Agricultural Emissions, PMF, Source Apportionment

## Abstract

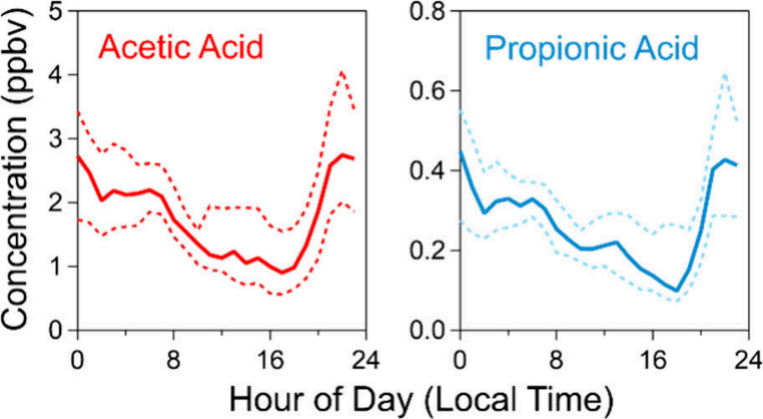

Gas-phase organic acids are ubiquitous in the atmosphere with mixing ratios of several species, such as formic acid and acetic acid, often as high as several parts per billion by volume (ppbv). Organic acids are produced via photochemical reactions and are also directly emitted from various sources, including combustion, microbial activity, vegetation, soils, and ruminants. We present measurements of gas-phase formic, acetic, propionic, pyruvic, and pentanoic acids from a site near Boise, Idaho, in August 2019 made by iodide-adduct chemical ionization mass spectrometry (CIMS). The site is adjacent to a major interstate highway and beyond the urban/suburban core is surrounded by national forests to the north and northeast and by farmland to the west and south. Maximum mixing ratios of formic, acetic, propionic, and pentanoic acid were typically near 10, 3, 0.4, and 0.2 ppbv, respectively. Observed daytime concentrations of these acids were mostly consistent with other studies, but concentrations were persistently the highest at night between 20:00 to 8:00 (local standard time). Such elevated nighttime concentrations are unlike most other reported organic acid measurements. Although there were times when organic acid concentrations were enhanced by mobile source emissions, the organic acid concentrations appear to be mainly controlled by noncombustion surface primary emissions. Source apportionment by positive matrix factorization (PMF) supports the importance of significant noncombustion, nonphotochemical emissions. Two agricultural surface sources were identified and estimated to contribute to greater than half of total observed concentrations of formic and acetic acid. In contrast to the other measured organic acids, but in agreement with all other reported measurements in the literature, pyruvic acid concentrations peaked during the daytime and were largely controlled by photochemistry.

## Abstract 2

In this study, measurements of several carboxylic acids near Boise, Idaho, are presented. Notably, the concentrations of carboxylic acids were elevated at nighttime, indicating a significant nearby surface source, likely from agriculture.

## 1 Introduction

Organic acids are a major portion of total gas-phase acids in the troposphere and are involved in the formation of secondary organic aerosols and thus have an impact on human health, visibility, and climate.^1−3^ Organic acids also affect the pH of cloud droplets and aerosol particles and as a result they can affect the health of terrestrial and aquatic ecosystems.^4,5^ Despite their importance, ambient measurements of organic acids are uncommon, and there are large gaps in our general understanding of their atmospheric sources and sinks. For example, discrepancies between measured and modeled carboxylic acid concentrations indicate uncertainties regarding the relative importance of sources, photochemical production, and/or atmospheric removal mechanisms.^6−9^ Chemical transport and photochemical box models have also suggested the potential for a missing sink.^9,10^

Formic acid and acetic acid are the most abundant organic acids in the atmosphere and are by far the most commonly reported in the literature. Ambient concentrations of these species often range up to several parts per billion by volume (ppbv).^11^ There has only been a small number of studies, however, that report three or more real-time gas-phase organic acid species.^12−15^ Reported gas-phase organic acid concentrations for > C2 acids (e.g., propionic acid) are typically lower than formic and acetic acid by at least an order of magnitude. These studies also show high correlation between many organic acid species indicating similar primary emission sources and/or chemical production mechanisms.

Organic acids have a variety of sources and relative source strengths that can vary greatly between acids for a given environment.^10^ Direct emissions of organic acids include combustion processes (e.g., from fossil fuels, biofuels, or biomass),^10,16−20^ soils,^10,21^ vegetation,^22^ and agricultural activities (e.g., cattle farming).^10,23^ Organic acids are also photochemically produced by the oxidation of VOCs.^11,24^ For example, the oxidation of biogenic VOCs is the largest global source of acetic acid.^25^ Oxidation pathways that lead to organic acid formation include reactions with ozone (O_3_), the hydroxyl radical (OH), and the hydroperoxyl radical (HO_2_). Ozonolysis of VOCs with a terminal alkene (e.g., isoprene, limonene) forms hydroxyalkyl hydroperoxides which can decompose into alkanoic acids.^26,27^ The reaction of peroxy acyl radicals with HO_2_ (or RO_2_; RO_2_ = organic peroxyl radical) can form organic acids. For instance, the reaction of the peroxy acetyl radical (CH_3_C(O)OO•) with HO_2_ yields acetic acid, per-acetic acid, and the methyl peroxyl radical CH_3_O_2_ in an approximate ratio of 15:41:44.^28−30^ Oxidation of secondary organic aerosol (SOA) relevant species may also act as a source of organic acids. For example, the aqueous phase photooxidation 2-methyltetrol, a particularly abundant component of SOA, yields formic and acetic acids.^31^ Heterogeneous OH oxidation of 2-methyltetrol sulfate derivatives have similarly been suggested as a source of formic and acetic acids.^32^

For many organic acids, atmospheric removal by reaction with OH is slow. For example, the reaction of acetic acid by OH has a rate constant of 7.4 × 10^–13^ cm^3^ molec^–1^ s^–1^,^33^ leading to an atmospheric lifetime with respect to OH-oxidation of ∼8 days assuming an average OH concentration of 2 × 10^6^ molecules cm^–3^. The major sink for most organic acids is thought to be atmospheric deposition.^24^ Removal by wet deposition is considered to be more important than dry deposition globally, though wet deposition rates depend greatly on the rate and frequency of precipitation.^24^ Carboxylic acids readily partition into aqueous aerosol and may subsequently undergo aqueous phase oxidation chemistry. This makes cloud processing a potential sink for various organic acids.^34^ In regions with little vegetation, uptake onto dust may act as an important sink, as organic acid ions (e.g., formate, acetate) can adsorb irreversibly to mineral aerosols.^35,36^

Although real-time, sensitive, and simultaneous measurements of various organic acids have become more commonplace in the last 15 years due to improvements in chemical ionization mass spectrometric (CIMS) techniques for atmospheric measurements, there are still few data sets that have focused on organic acids. Information on the concentrations, sources, and sinks of these acids will lead to a better understanding of their involvement in VOC oxidation in the atmosphere. In this study, an iodide-adduct CIMS was configured to quantify several organic acids at an established ambient air quality monitoring site near Boise, ID, USA in conjunction with other continuous regulatory and nonregulatory air pollutants. The major sources of the observed organic acids are investigated using supporting measurements and source apportionment modeling.

## 2 Materials and Methods

### 2.1 Measurement Location

**Figure 1:**
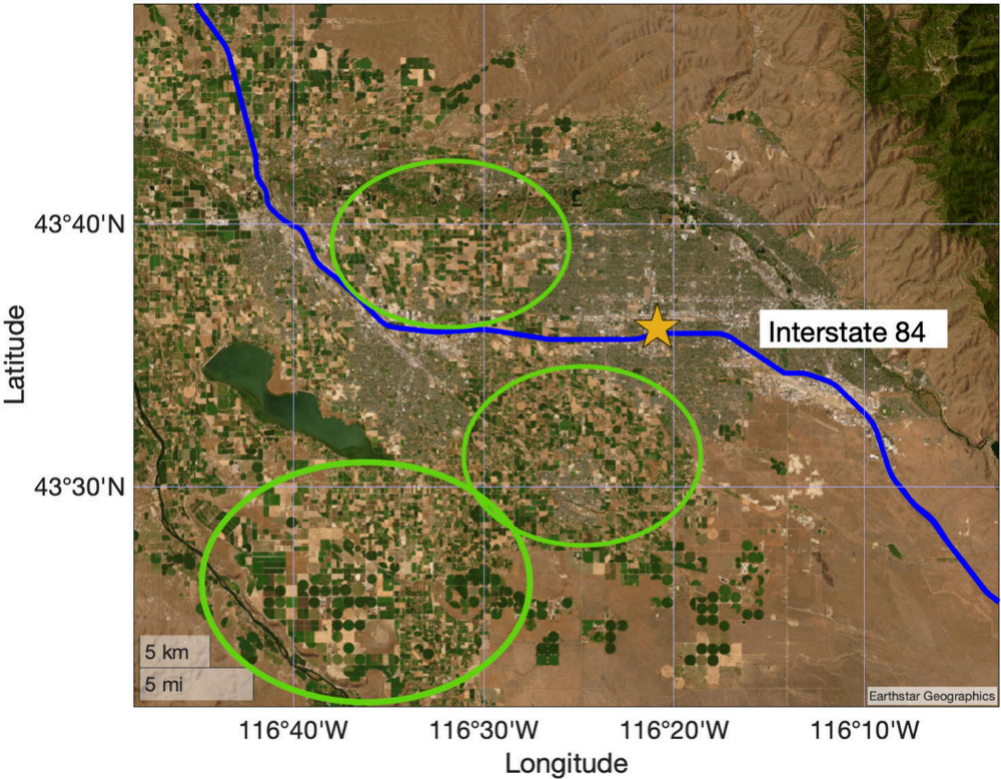
Map of the sampling site located in Meridian, ID, USA. Interstate-84 is indicated by the blue line. A few areas of land dominated by agricultural activity are circled. The satellite image was sourced from Earthstar Geographics hosted by Esri, and its use is permitted for academic publication.^37^

Measurements of organic acids and several other compounds were collected in Meridian, Idaho, during August 2019 in one of the Idaho Department of Environmental Quality’s (IDEQ) St. Luke’s Meridian (SLM) site (43.600703°N, −116.347952°W) monitoring shelters (see Figure 1). The IDEQ SLM site is the state’s U.S. EPA NCore Network monitoring site that comprehensively measures National Ambient Air Quality Standard (NAAQS) pollutants,^38^ and this site was host to a U.S. EPA Office of Research and Development wildland fire smoke monitoring study (2019–2022). The SLM site is approximately 10 km west of downtown Boise and 15 km east of Nampa. The measurement site was located 300 m North of Interstate 84 in an empty field behind a hospital. Beyond the SLM site’s directly surrounding urban/suburban core, the Boise metropolitan area is bounded by the Boise and Sawtooth National Forests to the northeast and east, Nampa to the west, and generally by agricultural areas (feedlots and dairy farms) and rural communities to the northwest and south. A few large swaths of agricultural areas are indicated in Figure 1. These areas are easily observable by satellite thanks to the distinctive circular shape produced by center-pivot irrigation and the contrast in color between the green irrigated lots and the natural Idaho terrain.

### 2.2 Organic Acid Measurements

#### 2.2.1 Chemical Ionization Mass Spectrometry

Organic acids were measured using a field deployable high-resolution Time-of-Flight iodide-adduct-CIMS (Tofwerk/Aerodyne Research, Inc.).^39,40^ The CIMS is described in full detail in the SI (Sect. 1.0). The CIMS notably contained a custom ion molecule reaction chamber (IMR) in which the reaction of sampled air with gas-phase iodide (I^–^) occurs. The IMR had a main sampling inlet and two opposing ports into which the reagent ion I^–^ and humidified N_2_ (for dilution) were added. The IMR lacked temperature control which led to significant deviations in sensitivity that are noted in Sect. 2.2.3. A schematic of the IMR and the mass spectrometer is shown in the SI. The instrument’s quadrupoles were set to “soft” voltage settings that were optimized to maintain rather than decluster iodide adducts.^39,40^ The resolving power was typically ∼4000 m/Δm during this experiment, less than the ∼5000 m/Δm often achieved in the laboratory.

The main organic acids of focus for this study are formic, acetic, propionic, pyruvic, and pentanoic acid and were detected as their adducts with iodide: I(HCOOH)^−^, I(C_2_H_4_O_2_)^−^, I(C_3_H_6_O_2_)^−^, I(C_3_H_4_O_3_)^−^, and I(C_5_H_10_O_2_)^−^, respectively. Analyte signals were interpreted using Tofwerk “TofWare” peak-fitting software. The software allowed for the deconvolution of overlapping peak signals caused by ions detected at similar mass-to-charge ratios. Most notably, detected ions of I(C_2_H_2_O_3_)^−^ and I(C_4_H_6_O_3_)^−^ were accounted for and have similar mass-to-charge ratios as propionic acid and pentanoic acid, respectively. These significant overlapping ion signals were often near or greater than their desired analyte-acid counterpart. Other minor ion peaks fitted by the software include urea (overlaps with acetic acid) and butyric acid (overlaps with pyruvic acid). Representative mass spectra showing deconvoluted signals at the nominal mass of each organic acid of interest are included and discussed in the SI (SI Sect. 2.0). No overlapping peaks were included for the interpretation of formic acid. This instrument was also calibrated to measure hydrogen cyanide (HCN) and nitrous acid (HONO) detected respectively as I(HCN)^−^ and I(HONO)^−^, and these measurements will the focus of an upcoming manuscript. HCN was calibrated using a standard gas cylinder post campaign yielding typical sensitivity of 0.04 ncps pptv^–1^. HONO was calibrated on site by the UV photolysis of water vapor in the presence of excess NO.^41^ Deconvoluted ion signals were normalized per 1 million ion counts of reagent ions (“ncps” = normalized counts per second), and the reagent ion signal was considered as the sum of the I^–^ and I(H_2_O)^−^ peaks. The total ion count rate was typically 3.2 million counts per second, and the typical ratio of I(H_2_O)^−^ to I^–^ was near 20%.

Isomers exist for all detected acid ions (besides formic acid), with glycolaldehyde serving as an example (having the same chemical formula as acetic acid). Such isomers may be detected by I-CIMS, though with differing sensitivities to their acid counterpart. The resulting acid concentrations (without considering isomers) are well correlated with formic acid (see Sect. 3.0), indicating minimal interference by possible isomers.

#### 2.2.2 Sampling and IMR Design

**Figure 2:**
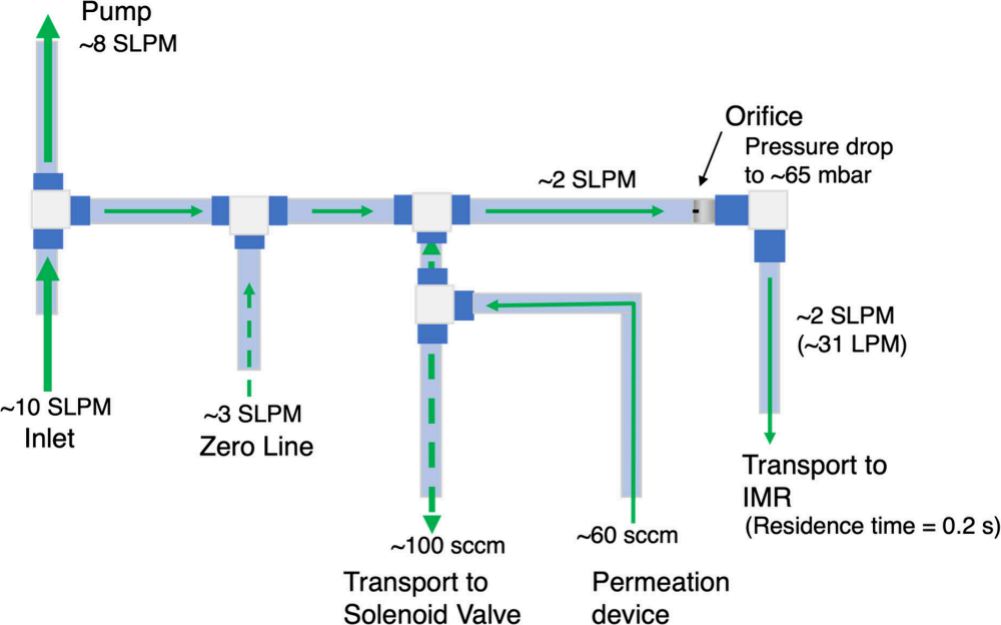
Schematic of the inlet manifold used for CIMS sampling. The flow path of the sampled air is indicated by solid arrows. Sampled air entered the inlet at ∼10 lpm, and a ∼ 2 slpm subsample was withdrawn from the major flow through a polytetrafluoroethylene (PTFE)-coated stainless steel orifice, which reduced the pressure to ∼65 mbar and increased the volumetric flow rate. From there, the air was transported to the CIMS IMR. Dashed arrows indicate periodic automated flows for standard additions of some analytes and instrumental zeros, introduced by overflowing the inlet manifold with (1) air from a cylinder or (2) scrubbed ambient air. Diagram not shown to scale.

The CIMS was housed in an air-conditioned monitoring shelter with the inlet manifold attached to the roof safety railing (inlet height of ∼3 m). The outdoor sampling inlet manifold is depicted in Figure 2. Air was sampled into 4 cm of 0.635 cm OD, 0.48 cm inner diameter (ID) perfluoroalkoxy (PFA) tubing at a flow rate of ∼10 L min^–1^ (LPM) as controlled by a small diaphragm pump. A 2 LPM subsample was withdrawn at a right angle from the inlet major flow line through a PFA T-fitting to reduce the amount of dust and insects drawn into the instrument sample manifold (via inertial separation). The manifold was configured with two more T-fittings, one for the periodic introduction of zero air for instrument background determinations and one for the introduction of organic acid (acetic, propionic, pyruvic, and pentanoic) standard additions. Two types of zero air were utilized during the study (i) commercially available scientific grade compressed gas cylinders and (ii) activated carbon scrubbed ambient air. We specifically apply the activated carbon-based backgrounds for the organic acid data presented in this manuscript. The IMR [H_2_O] value is nearly maintained during such zeros thereby avoiding drastic changes in sensitivity during an instrument background (see Sect. 2.2.3). Organic acid signals and IMR [H_2_O] values during a series of automated zeros and subsequent standard additions are provided in the SI (Figure S2) for reference. The organic acid standard additions were generated using laboratory-built permeation tubes that were held at constant temperature within a custom permeation tube holder subject to constant carrier flow of UHP grade N_2_. During sampling periods, the outflow of the organic acids from the permeation tubes was scrubbed through an activated carbon trap and vented. Standard addition periods were automated using a computer-actuated solenoid valve. The air then flowed through a 0.5 mm diameter stainless steel critical orifice internally coated with PTFE, decreasing the pressure by more than a factor of 10, a PFA elbow union, and through 3 m of 0.94 cm OD, 0.64 cm ID PTFE tubing into the CIMS IMR at a flow rate of 2 standard LPM (SLPM). The pressure in the IMR was held at 65 mbar and controlled by an Agilent Technologies (Santa Clara, CA, USA) model IDP-7 valve-throttled scroll pump. The resulting volumetric flow rate was 31 LPM, and the transit time from the pinhole outdoors to the IMR was calculated to be ∼200 ms. The relative humidity and temperature of the scroll pump exhaust were measured with a Vaisala (Vantaa, Finland) model HMP60 humidity probe to determine the water mole fraction in the IMR.

In contrast to many contemporary IMR designs, the initial pressure drop in our system occurs outside very close to the outdoor sampling point rather than in the IMR itself and the flow through the transit tubing is at reduced pressure rather than atmospheric pressure. This allows for relatively short transit times (200 ms) through the 3 m sampling inlet tube but without the need for a secondary pump or blower to attain the same volumetric flow rates at atmospheric pressure. More importantly, it allows us to easily zero the entire sampling system (inlet + instrument) by overflowing the entire inlet manifold with zero air since the total sampled flow rate was only 2 SLPM. The main disadvantage of this sampling method is the potential increase in diffusional wall losses of analytes within the sampling tube at reduced pressure.

#### 2.2.3 Standard Additions: Effect of Humidity and Temperature on Sensitivity

**Figure 3:**
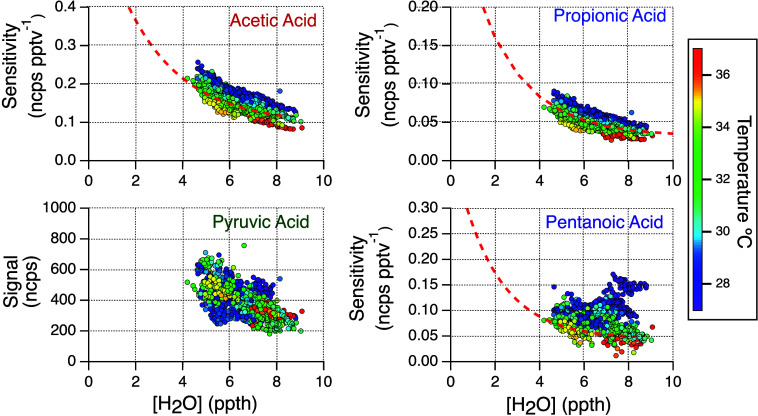
Standard additions reveal temperature and IMR water vapor effects on organic acid CIMS measurements. Each data point results from a single 60-s standard addition during the field measurements at the noted humidity and temperature. Plotted are the real-time sensitivities for acetic, propionic, and pentanoic acids and the addition signals (relative to ambient) for pyruvic acid signals. Markers are colored by the CIMS housing temperature during corresponding standard additions. Decreased sensitivities were generally observed at elevated temperatures and higher H_2_O mixing ratios. The dashed curves represent the laboratory-based calibration functions for experiments conducted at ∼30 °C with freshly prepared and characterized permeation devices.

The real-time sensitivity of the CIMS to various acids was monitored via automated standard additions (Figure 3) programmed to deliver consistent amounts of organic acids (acetic, propionic, pyruvic, and pentanoic) for a 1 min period every 10 min. A representative time series of automated additions is included in the SI (Figure S3) and notably shows a fast time response (<3 s) when switching between sampling and programmed standard additions. Standard additions led to increases of 34.8, 12.5, and 2.3 ppbv for acetic, propionic, and pentanoic acid, respectively. These concentrations were determined using permeation tube emission rates which were determined gravimetrically postcampaign (see SI Sect. 3.0). Due to difficulties in characterizing the pyruvic acid permeation tube permeation rate we elect to present pyruvic acid with a relative scale for this manuscript. Although no absolute concentrations are provided, the relative values presented have been corrected for the temperature and humidity dependence of the instrument. Standard additions for formic acid were not performed. Concentrations are therefore based on laboratory-based temperature and humidity-dependent calibrations (results included in the Sect. 4.0). The CIMS sensitivity to HCOOH was typically near 5 ncps pptv^–1^, which is similar to values reported for comparable instruments.^40,42^

Laboratory-based calibrations were performed for all measured acids greater than one year after the SLM field measurements, and calibration results are within reasonable agreement to the Figure 3 real-time standard additions and assist in confirming the tracked real-time sensitivities. Calibrations were conducted using a new set of permeation tubes that were freshly prepared and characterized gravimetrically. The full results of these calibrations along with greater detail of these calibration experiments are presented in the SI (SI Sect. 4.0).

The resulting instrumental sensitivity (i.e., calibration factor) determined from each standard addition is plotted in Figure 3. The median CIMS sensitivities to acetic, propionic, and pentanoic acid were 0.15, 0.06, and 0.08 ncps pptv^–1^, respectively. These sensitivity values for acetic and propionic acids are somewhat higher than literature values reported at similar water vapor concentrations.^40^ Differences in reported sensitivities for different CIMS users are caused mainly by different operating conditions, including IMR pressure, temperature, and instrument voltage settings. We are unaware of other literature values for the I-CIMS sensitivity to pentanoic acid. Its sensitivity dependence on [H_2_O] appears consistent with similar organic acids. Our nonstandard IMR design likely contributes to these differences as well.

Standard additions accounted for fluctuations in instrumental response caused mainly by changes in temperature and water vapor concentration (Figure 3). The effect of water vapor on CIMS detection of various analytes, including several organic acids, has been thoroughly discussed in the literature.^40,41,43^ The CIMS sensitivities (Figure 3) generally decrease with water vapor and are consistent with the general trends reported in the literature.^40^ The CIMS operated at a ∼ 10 °C temperature range throughout the campaign (∼27 to 37 °C) due to thermal management limitations experienced during the daytime. A decrease in CIMS sensitivities with temperature is easily observed, caused primarily by the temperature dependence of the various ionization reactions, and consistent with the trends reported by Robinson et al. (2022). There are few studies focused on CIMS ionization chemistry dependence on temperature. Example systems studied include SF_6_^–^ CIMS for detection of HNO_3_ and Br^–^ CIMS for the detection of HO_2_.^44,45^ The impact of temperature specifically on iodide CIMS-sensitivities has only recently been studied, and significant negative temperature dependences have been reported up to 6% per degree (°C) in a near linear fashion.^42^ The dependence of the CIMS sensitivity to acetic and propionic acids on temperature was most evident. For those acids, a ∼ 10 °C change results in an approximate 60%, or ∼6% °C^1–^, drop in CIMS sensitivity to acetic and propionic acid sensitivity at a constant humidity. This is comparable to the −5.3% °C^1–^ formic acid sensitivity dependence reported by Robinson et al. (2022).^42^ The temperature effect on CIMS sensitivity to pentanoic acid and pyruvic acid are less clear, though the sensitivity still appears generally greatest at cooler temperatures.

Maintaining IMR temperatures via heating or cooling greatly reduces any change in instrumental response. We therefore highly recommend, in agreement with Robison et al. (2022), that any IMR design incorporate temperature control in order to minimize such sensitivity-dependences on temperature while avoiding the need for intensive temperature–humidity characterization experiments. Since real-time formic acid sensitivity values were not tracked by standard additions, the CIMS sensitivity dependence on temperature was assumed equal to −5.3% °C^1–^ (i.e., that reported Robinson et al. (2022)^42^) at all IMR [H_2_O] values and scaled from the 31 °C reference temperature at the laboratory calibration was conducted. Furthermore, the laboratory-based sensitivity values applied to this data set were performed greater than 1 year after the measurements. For these reasons we choose a rather large formic acid uncertainty. The uncertainties for reported organic acid concentrations are discussed in the following section (Sect. 2.2.4).

#### 2.2.4 Measurement Uncertainties and Signal-to-Noise Ratios

The accuracy of presented concentrations is based on the uncertainties in the standard addition concentrations. The uncertainty associated from permeation tube permeation rates (and hence standard addition concentrations) was 2.0%, 4.8%, and 17.4% for the acetic, propionic, and pentanoic acid, respectively, based on the precision of the used analytical balance. The characterization of permeation tubes was conducted postcampaign, so we add in quadrature an additional 30% uncertainty to account for the possible drift in permeation rates. This additional 30% uncertainty also accounts for permeation tube mass loss not associated with the desired acid. Sensitivities determined by this permeation method may be biased low as a result leading to artificially high concentrations. The final measurement uncertainties are 30.1%, 30.4%, and 34.7% for acetic, propionic, and pentanoic acids, respectively. Standard additions were not performed for formic acid, and presented concentrations are rather based on calibration experiments conducted more than one year after the field measurements. We consider the accuracy as 50%.

The overall uncertainty for a given period is based on the combined precision of the ambient and background signals and the error associated with the temperature and humidity-dependent sensitivity (that is–dominated by its accuracy). These overall uncertainties vary throughout the data set due to changes in ambient signals along with fluctuations in sensitivity and background values. Signal to noise ratios (SNR) are used here to discuss precision and describe detection limits. The SNR equation is detailed in the SI (Sect. 5.0). Example ambient SNR values for a representative period of 09:30 MST on 18 August 2019 were 83,000, 1100, 77, 276, and 80 for formic, acetic, propionic, pyruvic, and pentanoic acid, respectively. For our instrument zeros, we found background noise to be greater than but dominated by theoretical shot noise for most acids (Figure S5). The detection limits for 15-s measurements of formic, acetic, propionic, and pentanoic acids were 2, 76, 21, and 23 pptv, respectively, calculated using the unnormalized CIMS acid signals during a representative humidity-matched zero (SNR = 2).

Calculations that only assume shot noise is present can result in erroneously low calculated detection limits if there are other sources of noise. The noise in the acetic acid signals was substantially higher than expected during backgrounds and ambient sampling (see Figure S5). For example, the manually calculated 76 pptv acetic acid detection limit is substantially greater than the 32 pptv theoretical shot noise-based detection limit. Analysis of the noise for the acetic acid peak signal and its nominal 187 *m*/*z* peak indicate that the high-resolution peak fitting process introduces substantial noise (see SI). Noise associated from ion fitting has been modeled and discussed in the literature.^46,47^ The noise in the ambient formic acid signals was also unexpectedly high–nearly twice the theoretical shot noise. Ambient formic acid signals were high, near 10^5^ cps (often 100 times greater than acetic acid signals), and these high signals may be a cause of the unexpectedly high noise (see SI), similar to the deviation in the integration errors for larger ion signals observed by Corbin et al. (2015).^48^ The noise of CIMS signals should be carefully considered for detection limits and error propagation.

### 2.3 Supporting Measurements

U.S. EPA gaseous supporting measurement instruments included (i) ThermoScientific (Franklin, MA, USA) model 48C gas filter correlation (GFC) Federal Reference Method (FRM) carbon monoxide (CO) analyzer, (ii) Teledyne API (San Diego, CA, USA) model T265 nitric oxide (NO) chemiluminescence O_3_ FRM analyzer, (iii) Teledyne API model T201 chemiluminescence ammonia instrument (NO, NO_2_, NO_*x*_, NH_3_), and (iv) Teledyne API model T360 M GFC carbon dioxide (CO_2_) instrument. All continuous gas analyzers were zero and span challenged weekly using a Teledyne API Model T700U dynamic dilution calibration system with a certified O_3_ photometer. EPA protocol certified gas standard cylinders diluted in ultrascientific grade zero air were used for CO, CO_2_, and NO_2_ instruments. No zero or span calibration adjustments were made during the study period as all zeros (0 ± 0.1 ppb) and spans (±0.5%) were within acceptance criteria. Multipoint span calibrations and converter efficiency tests were conducted monthly to ensure linearity and quantitative conversion. A meteorological tower operated by the Idaho Department of Environmental Quality was located at the site. Its relevant measurements include wind (velocity and direction), solar radiation, temperature, relative humidity, and barometric pressure.

### 2.4 Source Apportionment Modeling

Positive matrix factorization (PMF) analysis using the EPA’s PMF Model (Ver. 5.0)^49^ was used to investigate source apportionment of organic acids. A total of 10 gas-phase species were selected for PMF modeling. This includes the 5 organic acids measured by CIMS along with CIMS measured HCN. Supporting measurements of O_3_, CO, NO_*x*_, and NH_3_ were also input. To reduce PMF-model run times, the data set was averaged to a 15 min time base. Measurements of urea and HONO (both measured by the CIMS instrument) were considered for PMF analysis but ultimately excluded. Urea signals were minimal and often near that of the automated backgrounds with a lack of diurnal cycle observed. Temporal HONO data nearly matches NO_*x*_ observations but differs with the magnitude of changes in NO_*x*_. HONO is produced via multiple secondary chemistry processes. This leads to slight relative differences between HONO and NO_*x*_, and we consider HONO as an unnecessary PMF input. PMF inputs of individually measured NO and NO_2_ (as opposed to NO_*x*_) and Ox (Ox = O_3_ + NO_2_) were each considered but ultimately rejected. Lumped NO_*x*_ (NO + NO_2_) is advantageous as otherwise the PMF suggests individual factors for the two compounds.

The PMF software requires inputs of individual species uncertainties and detection limits. These inputs are specified in the SI (SI Sect. 6.0). The PMF model was initially run 20 times. The best solution was selected using a Q score that represents model robustness, and the results of this solution are shared in this manuscript. Error estimation methods were used to evaluate this solution. Bootstrapping was conducted using 100 bootstrap runs. For these bootstrap runs, > 98% of bootstrap factors were mapped to the corresponding factors of the base model run. This assists in indicating that a five-factor solution is appropriate.

## 3 Results and Discussion

### 3.1 Time Series and Diurnal Cycles

**Figure 4:**
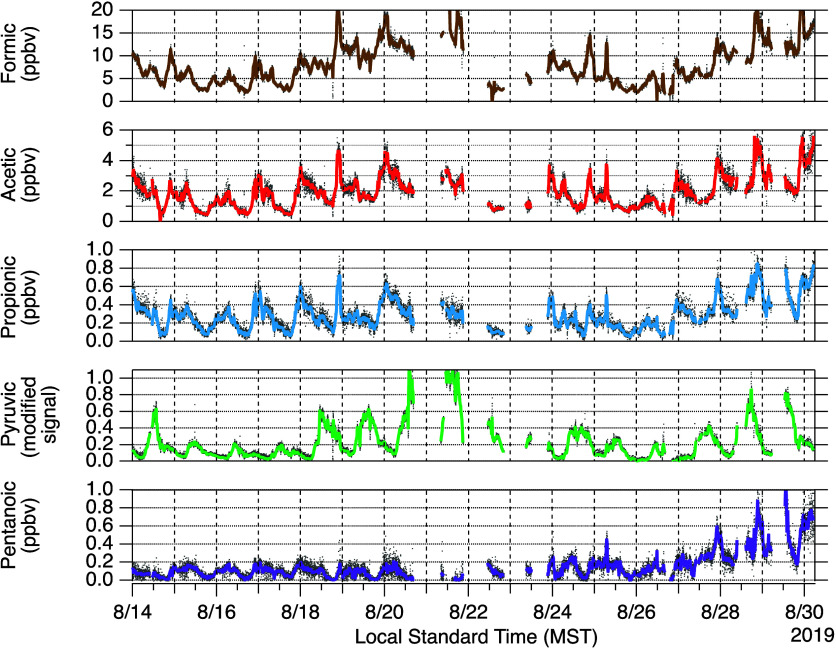
Time series data of organic acids. One- and 15 min averaged data are plotted, respectively, with gray and colored markers.

**Figure 5:**
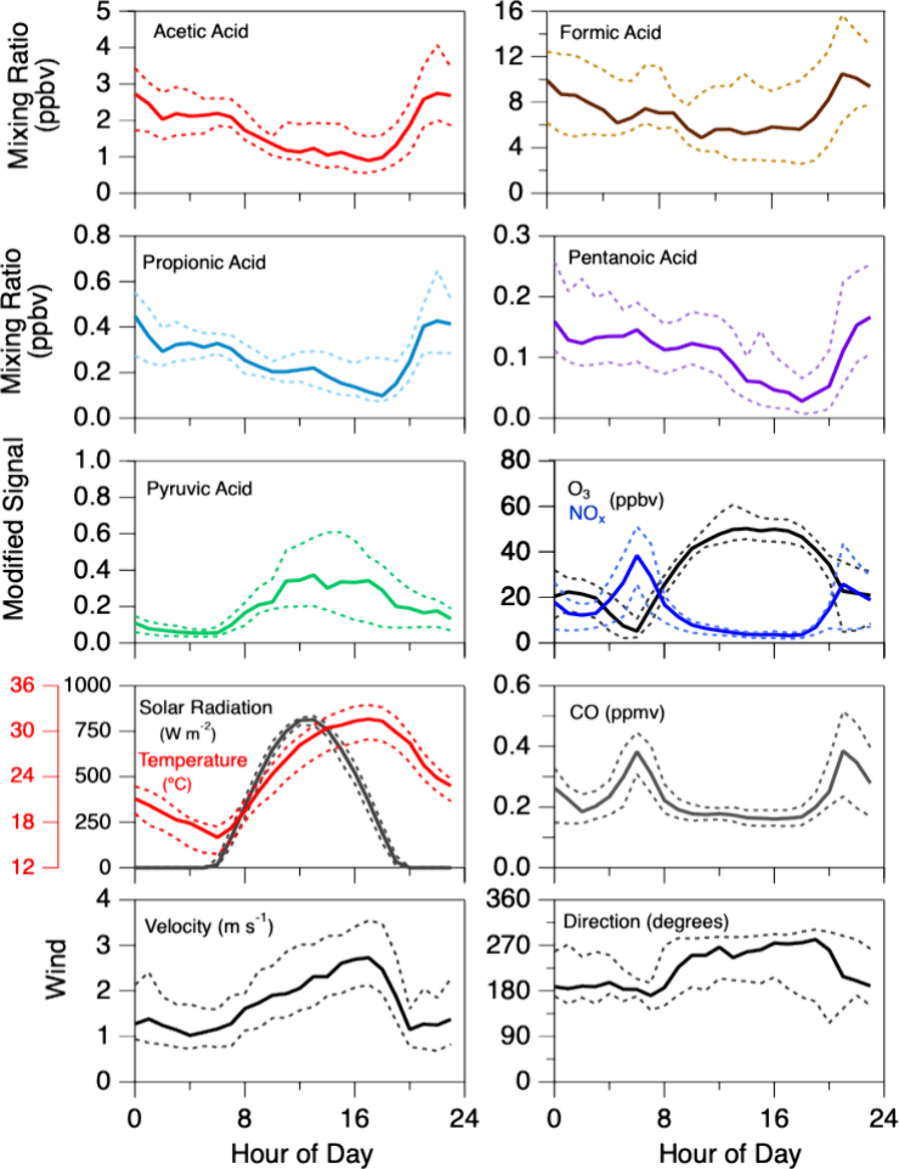
Diel profiles of organic acids, O_3_, NO_*x*_, CO, temperature, solar radiation, and wind. Median values are plotted in bold, and the 25th and 75th percentile data are indicated by dashed lines. For reference, a wind direction of 0° (and therefore 360°) represents a northerly wind. All times are local standard time.

**Figure 6:**
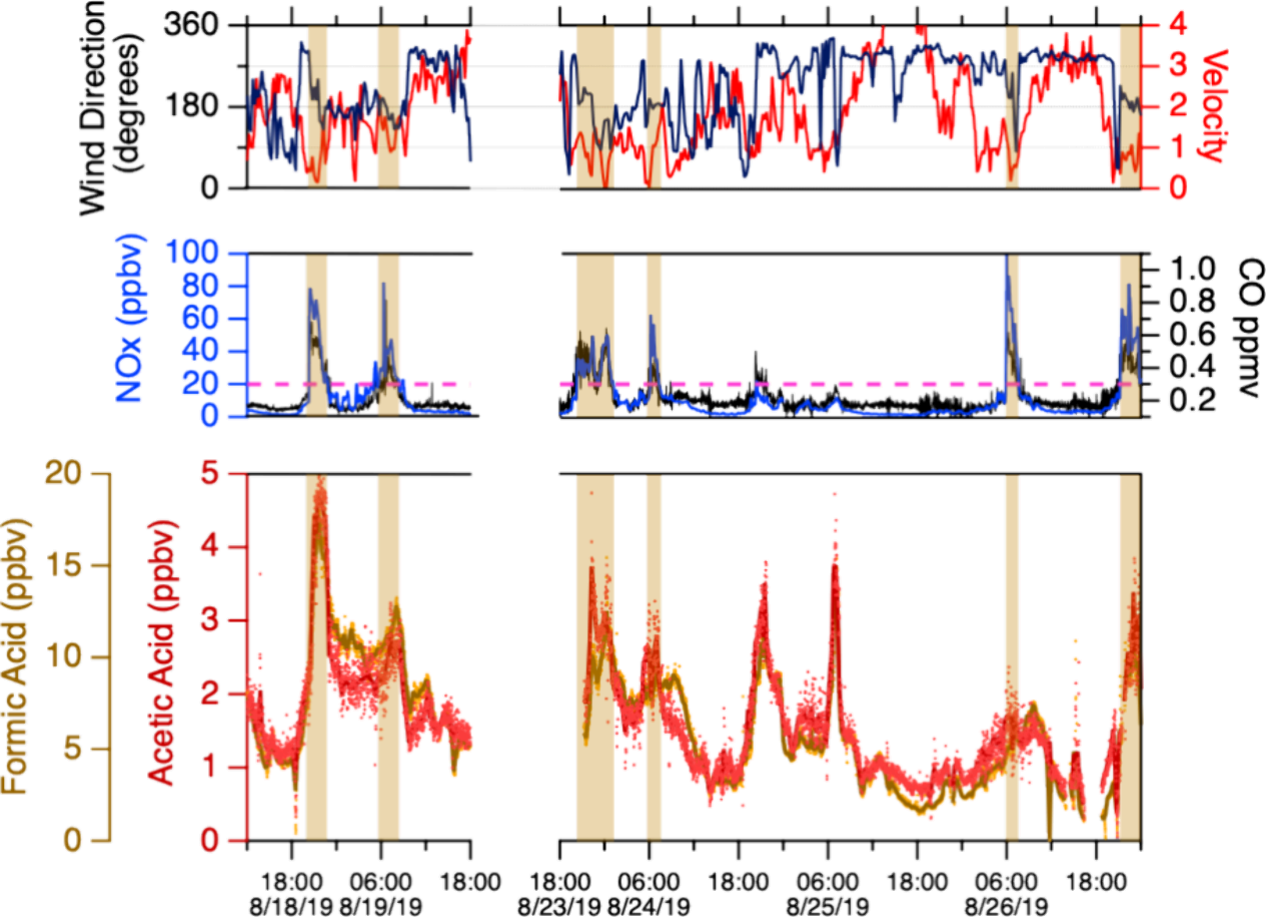
This representative time-series graph (local standard time) first shows a 30 h period where a shift to southerly winds causes strong highway influence (from I-84 located directly south) as evidenced by enhancements in NO_*x*_ and CO. Organic acid concentrations remained elevated overnight while NO_*x*_ and CO decreased to much lower values. The second portion of this time series is representative of times with minimal highway influence. Organic acids remain elevated at nighttime. Time periods with significant highway influence ([NO_*x*_] greater than 20 ppbv) are shaded.

Time series data for the measured acids are shown in Figure 4. Diel cycles of organic acids, as well as supporting measurements of NO_*x*_, O_3_, and wind, are presented in Figure 5. The measured values of NO_*x*_, O_3_, and CO are comparable to previously reported nonbiomass burning impacted observations made at this same site.^50^ NO_*x*_ was lowest during the daytime, typically below 5 ppbv, between 10:00 and 18:00 local standard time. Nighttime NO_*x*_ concentrations typically peaked near 20 ppbv, though greater values near 100 ppbv were occasionally observed. O_3_ values typically peaked near 57 ppbv, and nighttime values were typically near 20 ppbv. Values of NO_*x*_ and CO were observed to change with shifts in wind. Daytime winds were typically westerly (between 9:00 and 19:00 h) with the remainder of the time being southerly dominated. While this daytime-westerly and nighttime-southerly wind pattern is generally observed, there are lengthy exceptions (e.g., Figure 6).

Concentrations of the alkanoic acids (formic, acetic, propionic, pentanoic) were generally highest at night and declined during the day, reaching minimum values near 18:00. In contrast, pyruvic acid concentrations were highest during the day and lowest at night. This pyruvic acid diurnal cycle is consistent with the literature^12,13,51^ and known photochemical formation processes (e.g., ozonolysis of methyl vinyl ketone^52^ and photolysis of methylglyoxal^53^). Formic and acetic acids were the most abundant of the acids reported here, with concentrations ranging from 1 to 15 ppbv and 0.5 to 5 ppbv, respectively. Daytime formic acid concentrations measured at other locations are typically above 1 ppbv, sometimes exceeding 10 ppbv.^6,11,12,42,54−56^ Daytime acetic acid concentrations are typically reported in the literature to be 10% to ∼70% of the formic acid concentrations,^6,11,55,56^ consistent with our observations here.

Propionic acid was observed to range from 0.1 to 0.8 ppbv, with typical daytime concentrations near 0.2 ppbv. This daytime value is similar to that reported by Mattila et al., (2018) for a measurement site in Colorado Front where typical daytime concentrations were near 0.1 ppbv but lower than the daytime values of several ppb observed in the urban outflow of Los Angeles.^12^ Pentanoic acid had the lowest concentrations of the acids reported here, rarely exceeding 0.2 ppbv at night and roughly an order of magnitude lower during the day. Pentanoic acid concentrations have been reported to range up to 30 pptv, peaking during daytime.^13^

Diel profiles for organic acids reported at other locations typically show peak concentrations midday.^12,13,42,55^ The organic acid diel cycle shown here is unlike most other reported organic acid diel cycles (besides pyruvic acid) as the concentrations were greatest overnight. A similar observation was made for a North China Plain-based study where several acids were greatest overnight,^15^ consistent with our observations. The greatest source of pyruvic acid at our site is likely photochemistry as it is produced by the ozonolysis of biogenic compounds (e.g., O_3_ + methyl vinyl ketone).

The consistency between formic, acetic, propionic, and pentanoic acid strongly suggest that these acids have common sources. Formic, acetic, and propionic acids are each highly correlated with each other (Pearson rank^57^ (PR) > 0.70) thus strongly suggesting common sources. Pentanoic acid was highly correlated with propionic acid (0.71) and moderately correlated with formic (PR = 0.34) and acetic acid (0.49). The elevated concentrations of acids observed at night (Figure 5), when the mixing height (not measured) was undoubtedly low, strongly suggest that a significant direct surface emission source is present. Since the combustion products NO_*x*_ and CO are highest at night as well it would be tempting to assign vehicles as a significant source of the alkanoic acids. Close examination of the time series of these compounds (Figures S7 and S8), however, indicates that vehicles are at most a minor source of alkanoic acids. Consider the period shown in Figure 6. The NO_*x*_ and CO concentrations rapidly increased when the wind direction changed abruptly from northwesterly to southerly at 20:00 on 18 August. Concentrations of the alkanoic acids did not increase during this wind change, but only gradually increased starting 10 min later. The organic acid I-CIMS data is definitely time aligned with the supporting CO and NO_*x*_ measurements: HONO and HCN concentrations measured by the same I-CIMS (the focus of an upcoming manuscript)^41^ closely track the CO and NO_*x*_ concentrations in time. The observed HONO/NO_*x*_ emission ratios are approximately 1%, consistent with literature values.^58^ Furthermore, we would only expect modest enhancements in organic acid concentrations from vehicular emissions of approximately 850, 200, and 20 pptv of formic, acetic, and propionic acid, respectively, during a typical highway influence period assuming diesel emission factors^16,59^ of 130, 30, 5 mg kg^–1^ and the 20 ppmv CO_2_ increase observed here.

After this strong highway influence period, NO_*x*_ and CO decreased to much lower values ([NO_*x*_] < 5ppbv and [CO] < 150 ppbv) around 23:00 while all of the alkanoic acids considerably decreased in concentration but remained elevated compared to daytime concentrations. A second three-day period from 23 August to 26 August is included in Figure 6. The 23 August air was characterized by slow southerly winds and hence periods of strong highway influence. The following 2 days were characterized by westerlies. The westerly dominated nights were mostly free of vehicular emissions (as evidenced by near background NO_*x*_ and CO) but had levels of organic acids similar to highway-influenced nights. Propionic and pentanoic acids are not included in Figure 6, but their temporal trends are mostly consistent with the formic and acetic results.

These observations are consistent with the presence of a noncombustion, surface source of alkanoic acids in the region. Concentrations were highest at night due to the decreased vertical mixing (low mixing height), as commonly observed for most compounds emitted at the surface (e.g., CO and NO_*x*_ in urban areas). The source is almost certainly agricultural in nature since the site is surrounded by farmland and cattle grazing (Figure 1). These surface emission rates certainly vary with conditions (e.g., temperature strongly influences soil acid emissions^25^) though its impact on surface concentrations would be highly dependent on vertical dilution. Assuming a constant source emission rate, the surface source would have a greater contribution to observed concentrations during nighttime (i.e., low mixing heights) and play a smaller role during the daytime with effective vertical dilution.

### 3.2 PMF Analysis

The five factor PMF results are presented in this section. While the PMF technique is useful for interpreting the time series and semiquantitatively estimating the source apportionment, this technique certainly has limitations given it does not explicitly account for chemical losses. The model predicted values generally compare well with the measured data points. Linear regression analyses (i.e., model prediction vs observed data) for supporting measurements NO_*x*_, O_3_, and NH_3_ had slopes and r^2^ values of >0.98 and >0.99, respectively. Model predicted CO compared somewhat lower with a slope = 0.92 and r^2^ = 0.92. The HCN prediction-observation regression yielded a poor r^2^ value of 0.38 and slope of 0.54. Closer inspection of the predicted HCN time series, however, shows close alignment with observations for two multiday periods. A > 200 pptv offset between predicted and observed values persisted for a 4-day period likely caused by a shift in the background atmospheric HCN concentration. This HCN discrepancy resulted in the low comparison (i.e., slope) and worse correlation (r^2^).

The model predicted values of the organic acids also generally compare well with observations. Predicted vs observed regressions of acetic, propionic, and pyruvic acids values had r^2^ values >0.92 and slopes of 0.98, 0.89, and 1.00, respectively. Formic and pentanoic acids had weaker correlations with r^2^ values of 0.81 and 0.50, respectively. The model predictions of formic and pentanoic also compare low to those observed with regression slopes found equal to 0.76 and 0.22. The low pentanoic acid predictions may be attributed to the increased PMF input uncertainty.

**Figure 7:**
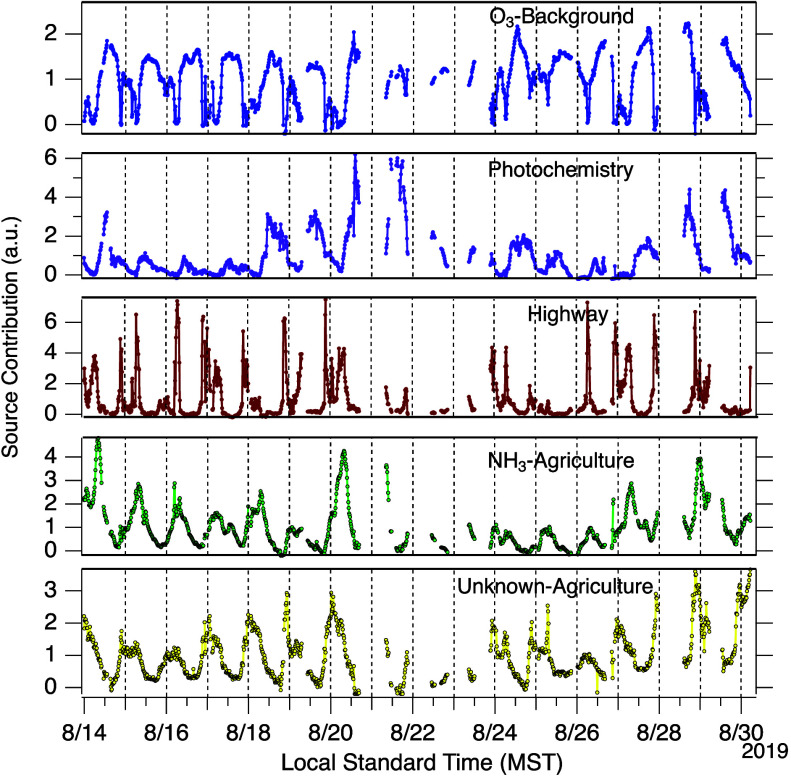
Source contributions for five factor PMF solution.

**Figure 8:**
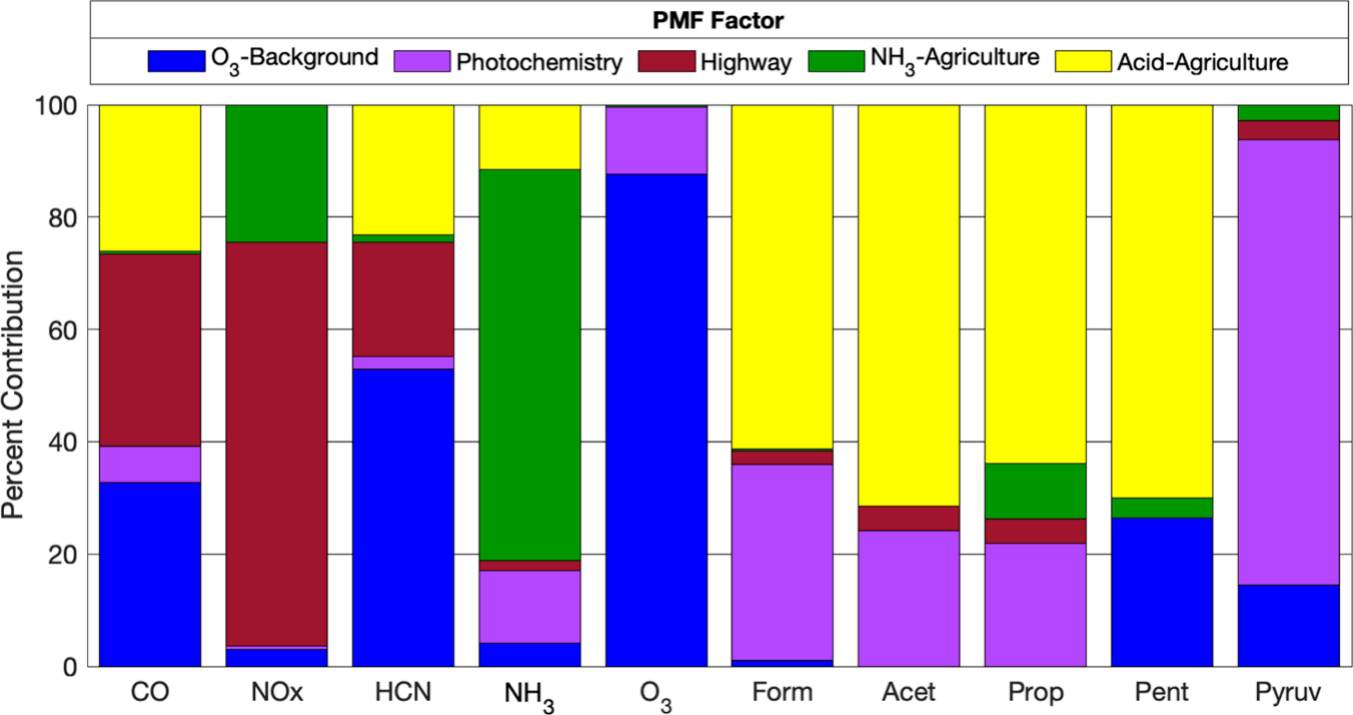
Overall factor contributions (percentage of species sum) for model predicted species. Titles of organic acid species are abbreviated (e.g., “Acet” represents acetic acid).

Time series data of the five PMF factors are shown in Figure 7. Diel profiles of these factors are included in the SI (Figure S6). We assign labels to these factors as ‘O_3_–Background’, “Photochemistry”, “Highway”, ‘NH_3_–Agriculture’, and “Acid-Agriculture”. Overall contributions of these factors to the PMF predicted concentrations are shown in Figure 8. The ‘O_3_–Background’ factor accounts for most ozone observations, which rarely exceeded background values typical of the Western US.^60^ Due to this factor’s association with O_3_, its contribution was predicted as generally greatest during the day (when O_3_ was highest) and lowest at night. This factor also accounts for portions of nonzero daytime values of other measured species (e.g., CO, HCN). The “Photochemistry” factor describes a portion of daytime O_3_ values (roughly all concentrations greater than 45 ppb) as well as >75% of all predicted pyruvic acid. As a result, this factor has a minimal contribution during nighttime. The “Highway” factor was characterized by its minimal daytime influence with periodic spikes observed at night associated with emission tracers of NO_*x*_, CO, and HCN. The spikes in NO_*x*_ and CO depicted in Figure 6 caused by sampling highway influenced air are, for example, captured by this “Highway” factor. The factor identified as ‘NH_3_–Agriculture’ contributed to ∼70% of NH_3_ predictions and is identified as an agricultural factor because of the association of NH_3_ with agriculture.^61−63^ This factor generally had little influence in the evening (minimum contribution near 19:00 h MST) after which this factors contribution would build and persist into the morning and daytime with a typical peak factor influence occurring near 7:00 h MST. The final factor is identified as “Acid-Agriculture”. This factor has a unique diurnal profile from the ‘NH_3_–Agriculture’ factor with minimal influence during day and greatest influence at night observed. We assume that this factor is agricultural in nature due to the site’s proximity to agricultural lands, this factor being unique from the highway source (i.e., noncombustion related), and its >12% estimated contribution to NH_3_. The existence of multiple agriculture factors is unsurprising given the various types of agricultural areas nearby (e.g., soil vs livestock), their variable distance from the measurement site, and that the dependence of these emission rates on temperature, humidity, and soil conditions are not identical.^25^ Difference in gas-particle partitioning for different compounds emitted by agriculture (e.g., ammonia and ammonium nitrate) may also explain why a single PMF factor is not adequate. This factor is closely associated with alkanoic acid predictions.

The PMF solution suggests a similar conclusion as the time series analysis from section 3.1 regarding the sources that control the concentrations of the alkanoic acids (i.e., the measured acids excluding pyruvic). For formic, acetic, and propionic acids, this “Acid-Agricultural” factor contributed between 64 and 71% to predictions. Other factors had similar contributions to the formic, acetic, and propionic predictions. The ‘NH_3_–Agricultural’ factor contributed <10%, the photochemistry factor contributed 22–35%, and the highway factor contributed <5% overall. The pentanoic acid PMF results, in contrast, had minimal contributions from the highway and photochemistry sources. Approximately 70% of pentanoic acid predictions were associated with the “Acid-Agricultural” factor and ∼4% associated with ‘NH_3_–Agriculture’, with the remaining 26% of predicted values associated with ‘O_3_–Background’ (i.e., corresponds with the background daytime values).

Though the alkanoic acid concentrations were highest at night and the PMF results indicated that the factors identified as agricultural were the most important, this should not be interpreted to mean that the agricultural emission source is greater than the photochemical source. The photochemical source produces these acids throughout the relatively high atmospheric mixing height during the day, whereas the surface source only leads to high concentrations observed at the surface because of the low nocturnal mixing height. Vertically resolved measurements would likely indicate a significant gradient observed at night consistent with those observed for other compounds emitted at the surface.^6^

### 3.3 Implications

Primary agricultural emissions (including soils) appear to be an important source of alkanoic acids in Boise, ID and were responsible for the unique diel cycle observed that show elevated concentrations persisting into the night. There are only a limited number of studies that focus on agricultural emissions of organic acids, specifically formic and acetic acid.^21,23,25^ Organic acids are occasionally used as tracers of air mass history since they are products of VOC oxidation,

and further research would be beneficial for quantifying their budgets and contribution to atmospheric chemistry. A more direct emission study, such as examining soil fluxes, would provide valuable insight into the importance of direct emissions for various species. Future observations of lower volatility organic acids (i.e., greater than 4 carbons) would benefit from speciated particle-phase measurements which would offer useful information on gas-particle partitioning.

## Data Availability

EPA supporting monitoring data can be found at https://catalog.data.gov/dataset/epa-sciencehub.

## Supporting Information Available

Greater detail of the CIMS: schematics, flow rates, peak fitting, automated zeros, and standard additions; permeation tubes: characterization and comparison to laboratory-based calibrations; analysis and discussion of CIMS noise; parametrization of PMF source apportionment model and PMF output diel profiles; time-series analysis for example highway influenced period (PDF)
