# Supplementary material for: Noncombustion Emissions of Organic Acids at a Site near Boise, Idaho
Source: ACS EST Air. 2024 Nov 27;1(12):1568–78. doi: 10.1021/acsestair.4c00138 (PMC11650544; doi:10.1021/acsestair.4c00138)
Supplement: Supplementary file 1 — ea4c00138_si_001.pdf [file ea4c00138_si_001.pdf]

Supporting Information

Non-combustion Emissions of Organic Acids at a site near Boise, Idaho

Andrew J. Lindsay†, Brigitte M. Weesner†, Kyle Banecker†, Lee V. Feinman†, Russell W. Long‡, Matthew S. Landis‡, *Ezra C. Wood† (ew456@drexel.edu)

†Department of Chemistry, Drexel University, Philadelphia, PA, 19104 USA

‡US EPA, Office of Research and Development, Research Triangle Park, NC, 27711 USA

ᘓ Current Address: Department of Chemistry, University of Colorado Boulder, Boulder, Colorado, 80309, United States

1.0 Description of CIMS

The high-resolution Time-of-Flight iodide-adduct-CIMS (Tofwerk/Aerodyne Research, Inc.) was used for this work. The instrument comprises a custom ion molecule reaction chamber in which analyte molecules react with iodide (I^-) reagent ions, a series of intermediate transfer stages through which the ion products of the ion-molecule reactions are guided by quadrupoles through several pressure drops, and a reflectron time-of-flight (ToF) mass spectrometer with a microchannel plate (MCP) detector (Fig. S1).

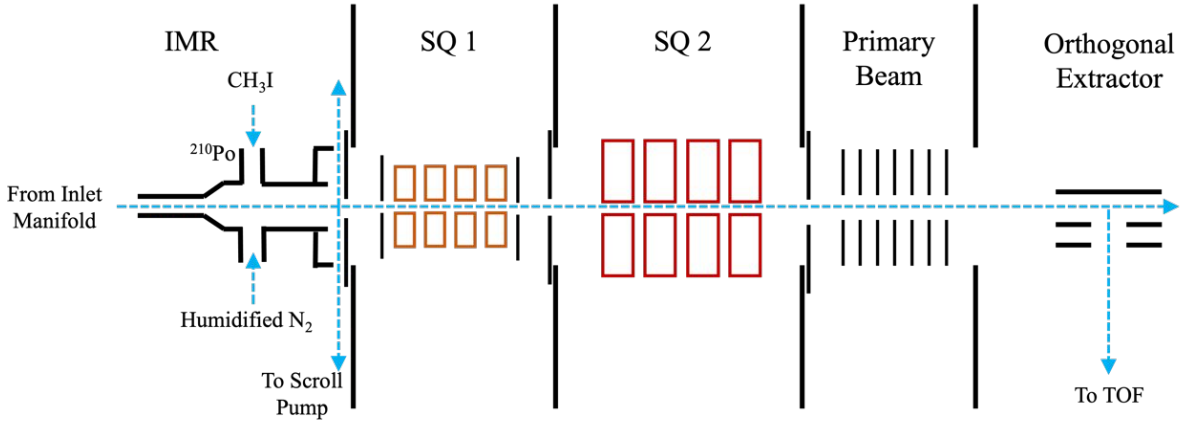

Figure S1 Schematic of the IMR and mass spectrometer (not shown to scale). The initial pressure drop is further upstream (see Fig. 2 of main text). IMR = Ion Molecule Reactor, SQ = Segmented Quadrupole.

The custom IMR was designed and machined in-house and consisted of a stainless-steel tube (10.8 cm length and 2.54 cm outer diameter (OD)) with two opposing ports on the side both located 3.8 cm downstream of the IMR entrance. A fluorinated ethylene propylene (FEP) tube (OD = 2.22 cm) that was altered to permit flow from the two opposing IMR ports was inserted into the main stainless-steel tube to act as a nonreactive internal coating. The iodide reagent ion was introduced via one of the side ports and was prepared by exposing ~170 ppbv of gas-phase $CH_3I(g)$ in a flow of 2 SLPM N_2 to alpha particles emitted by an NRD, LLC (Grand Island, NY, USA) model P-2021 ^{210}Po-based ionization source. Humidified nitrogen (0.1 SLPM), prepared by flowing UHP nitrogen through a bubbler containing HPLC-grade water, was introduced via the opposing side port (0.64 cm OD) in order to reduce the variability of the humidity in the IMR. A portion of the air exiting the IMR was sampled through a stainless-steel pinhole (0.5 mm diameter) into the series of quadrupoles and ion optics that guide the ion-molecule adducts to the ToF analyzer. The quadrupoles (as mentioned in the main text) were set to “soft” voltage settings that were optimized to maintain rather than decluster iodide adducts.[1,2] In the field our resolving power was ~4000, less than the typical value of ~5000 that we have obtained in the laboratory.

2.0 Peak Fitting of Ambient Data

The detected peaks associated with acetic acid, propionic acid, pyruvic acid, and pentanoic acid each have interferences from ions of a similar mass-to-charge ratio during ambient sampling (see Fig. S2). The Tofwerk AG (Thun, Switzerland) peak fitting software *TofWare* was used to

identify and computationally separate the peak area of each desired analyte ion. For acetic acid, we account for the presence of urea (detected as $ICH_4NO_2^-$). The propionic acid peak has an interference from a lighter weight ion that is most likely the iodide adduct of glyoxylic acid (i.e., detected as $IC_2H_2O_3^-$). Pyruvic acid is somewhat obscured by the presence of a higher molecular weight ion, the identity of which is most likely butanoic acid (detected as $IC_4H_8O_2^-$). Pentanoic acid is obscured by the presence of $IC_4H_6O_3^-$ (i.e., iodide adducts of $C_4H_6O_3$), for which many possible isomers exist (e.g., 3-oxobutanoic acid).

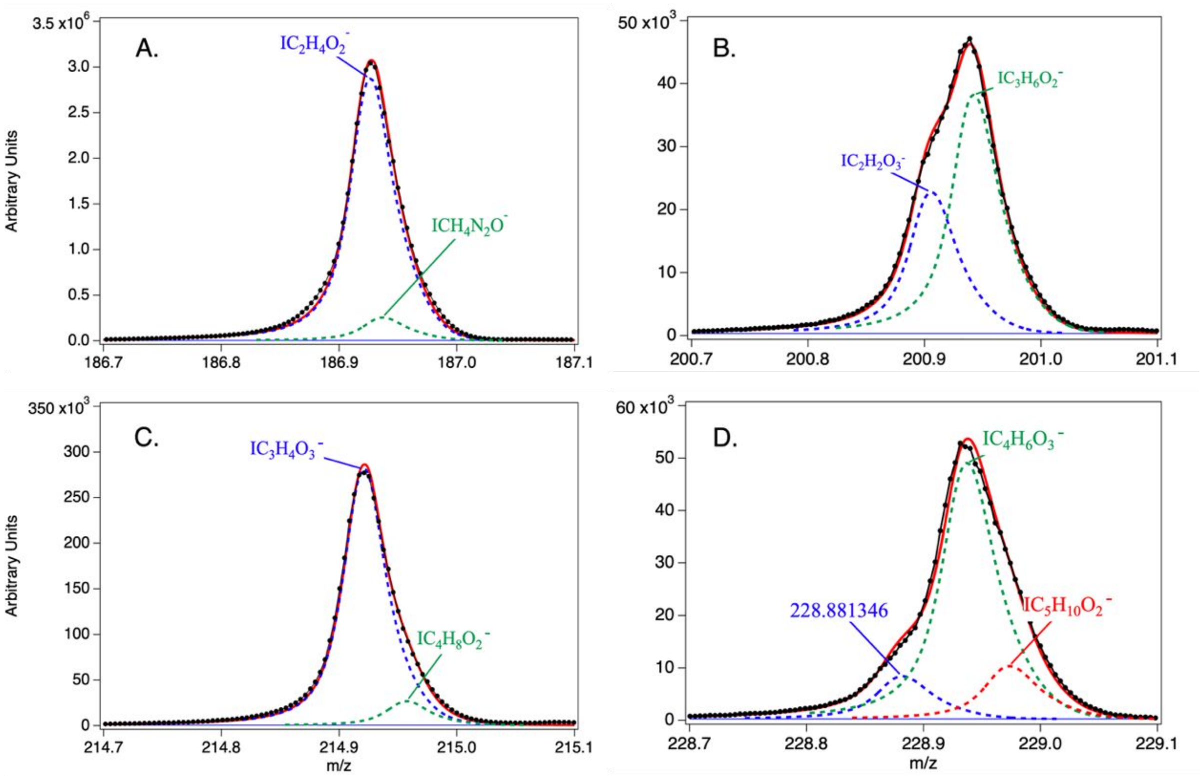

Figure S2 Fitted peaks for detected iodide adducts of acetic acid ($IC_2H_4O_2^-$; panel A), propionic acid ($IC_3H_6O_2^-$; panel B), pyruvic acid ($IC_3H_4O_2^-$; panel C), and pentanoic acid ($IC_5H_{10}O_2^-$; panel C) for a representative period. Interfering ions that were included in the fitting of these peaks are shown. The black markers represent the observed spectrum, the dashed lines represent the peaks associated with single ions, and the red line indicates the sum of all fitted peaks.

3.0 Automated Standard Additions and Zeros

A permeation device held four organic acid permeation tubes at constant temperature. Twenty sccm of N_2 flowed through the device constantly and was periodically introduced into the sample manifold throughout this experiment (i.e., automated standard additions were performed). The permeation tubes used during this experiment were laboratory-built and prepared using pure (>99.9% purity) organic acids. The rates of permeation for these tubes were characterized post-experiment (see Table S1) via a multitude of mass measurements over an extended period of use.

Table S1. Summary of organic acid permeation rates and the concentration delivered to the CIMS IMR during standard additions.

	Permeation Rate (ng min^{-1})	Concentration Delivered to IMR (ppbv)
Acetic Acid	160	34.8
Propionic Acid	71	12.5
Pentanoic Acid	18	2.3

A representative time series of CIMS data is provided in Fig. S3. CIMS signals (counts per second) for detected acids are shown normalized per million reagent ions (i.e., normalized counts per second or 'ncps'). The representative time series includes three standard additions. Each standard addition is followed by two automated zeros. This pattern and timing of standard additions followed by two zeros (one minute of additions/zero every 10 minutes of sampling) was mostly maintained throughout this campaign. Panel B of Fig. S3 shows a zoomed in section of data to show the time response of additions and zeros. Notably, the additions had a 2-3 second

transition period at the start of the automated addition and minimal tailing (~2 s) when switching back to the sampling mode.

The first zero conducted after the standard addition shown in Fig. S3 was performed by pumping ambient air through a reactor containing activated carbon. Water vapor within the CIMS IMR (also shown in Fig. S3) during this type remains similar to when sampling ambient air for this type of zero. A direct flow of cylinder air to the sample inlet was automated for the second zero after the standard additions. For this type of zero, water vapor within the IMR is greatly reduced and is thus not representative of the sampling period. We therefore use our filter-based zeros for the interpretation of the CIMS data presented in this manuscript. The interpolated background signal between neighboring zero periods was subtracted from the intervening sampling data to account for any drift in the instrument background values.

There were difficulties characterizing the pyruvic acid permeation rate. The implementation of our determined pyruvic acid gravimetric permeation rates resulted in standard addition concentrations that yielded sensitives that were low by more than a factor of 10 compared to the literature.[1] Applying such sensitivities produced unreasonably high concentrations. Since the field pyruvic acid standard additions were consistent (i.e., mainly varying due to temperature and humidity), we determine only a relative sensitivity to pyruvic acid and apply this to the data. As a result, unitless pyruvic acid data is presented in the manuscript but is corrected for fluctuations in sensitivity caused by humidity and temperature. Post-campaign laboratory calibrations of organic acids (discussed in the following section) further showed characterization of pyruvic acid to be difficult. We conclude that implementation of the pyruvic acid calibration via gravimetric analysis of a permeation tube is likely not viable.

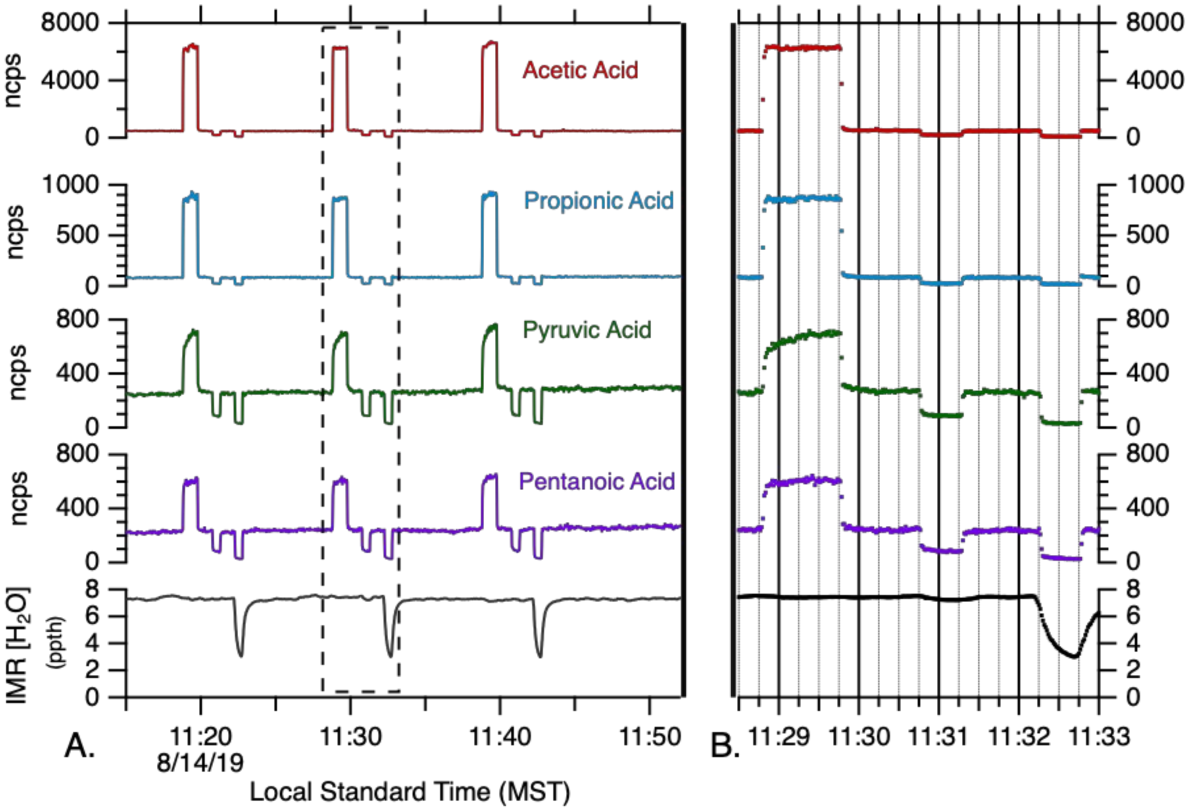

Figure S3 Normalized 1 Hz signals for the detected ions associated with organic acids for which perm tube-based standard additions were performed for a representative time period. For this section of data, automated standard additions were followed first by a filtered, activated carbon-based zero and second by a cylinder air-based zero. Water vapor mixing ratios in the CIMS IMR are plotted to show its minimal change during zeros and standard additions. Panel B shows a zoomed in time series of a specific addition cycle (boxed area of panel A).

4.0 Laboratory Calibration of Acids

Additional calibration experiments were conducted more than a year after the Boise field study. The goal of these additional, more recent calibrations was to confirm the real-time sensitivities observed field observations.

New and freshly prepared permeation tubes were characterized gravimetrically (Table S1). Permeation rates were determined by recording masses several times over a multi-week period of constant use (i.e., held at a constant temperature of 40 ^{o}C with constant N_2 flow). These permeation

tubes were prepared using pure acids (formic: Fisher Chemical, ≥99%; acetic: Fisher Chemical, ≥99%; propionic: Fisher Chemical, ≥99%; pyruvic: Fischer, 98%; pentanoic: Alfa Aesar, ≥99%).

Table S2. Rates of permeation for recently prepared permeation tubes and the resulting concentrations delivered to the CIMS IMR.

	Permeation Rate (ng min^{-1})	Concentration Delivered to IMR (ppbv)
Formic Acid	105	26.7
Acetic Acid	156	34.8
Propionic Acid	9.4	1.5
Pentanoic Acid	2.3	2.1

I-CIMS sensitivity results are shown plotted in Fig. S4. The calibration procedure for all acids was performed simultaneously. Acids were added continuously by flowing 80 sccm N_2 through the permeation tube holder, then diluting into a humidity-controlled flow of 2.1 LPM cylinder air that enters the CIMS IMR. The humidity of the main sample flow was maintained by mixing a flowrate of humidified air (i.e., cylinder air bubbled through water) with a flowrate of dry air, and the combined flowrate was vented to 2.1 LPM (i.e., the sample flowrate of the CIMS IMR) prior to the acid addition. Acid background signals were obtained for each humidity setting by temporarily diverting the acid flow with a three-way solenoid valve. The calibration results (i.e., instrument sensitivities) and the effect of water vapor generally agree with the results described by Lee et al., (2014).[1] The sensitivity of I-CIMS to pentanoic acid has not been described elsewhere in the literature, and these results reveal that I-CIMS is less sensitive by nearly an order magnitude compared to the most similar acids of acetic/propionic here. A second-degree polynomial was used to fit the formic acid sensitivity-dependence on water vapor. Exponential

equations with a y-offset were used to model all other organic acid sensitivity dependences on water vapor.

A separate vapor pressure-based calibration technique was attempted for all organic acids. These calibrations involved bubbling N_2 through an acid solution followed by dilution into a larger air flow. Acid concentrations delivered to the IMR were based on their literature vapor pressures and the measured dilution ratio. This technique resulted in lower sensitivities compared to our permeation tube-based sensitivities (often by a factor of 10) and to literature values (e.g., Lee et al., (2014)[1]). These calibration results would yield unreasonably high concentrations if applied to our dataset. This method is likely not viable for the calibration of organic acids and would require a separate technique to determine outflow concentration.

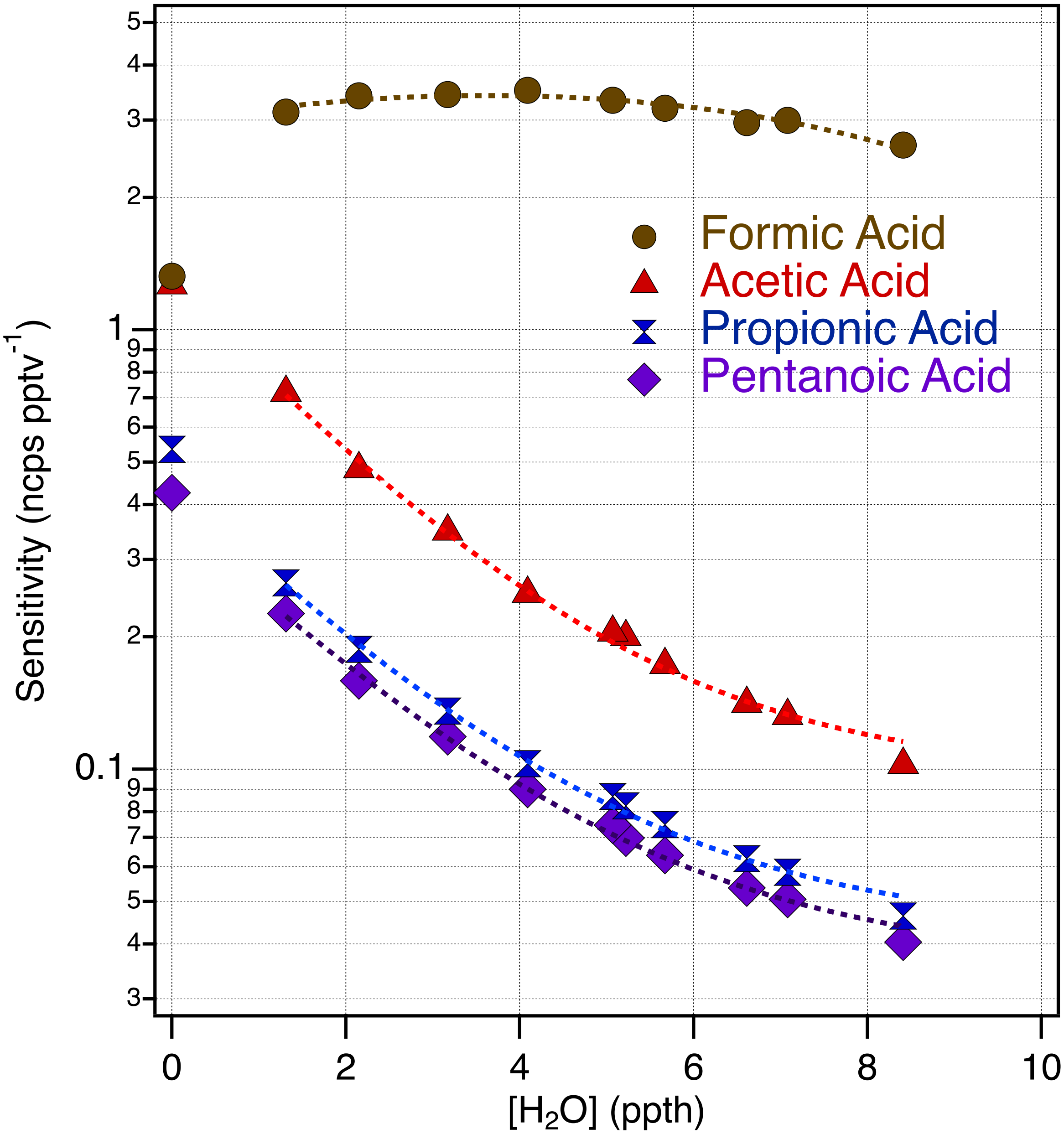

Figure S4 Laboratory results for CIMS organic acid sensitivity to organic acids. The acids of interest were calibrated simultaneously using freshly prepared and characterized permeation tubes. The CIMS temperature was ~30 ^{o}C, and the CIMS was operated to match conditions (e.g., IMR pressure, quadrupole voltages, single ion count, etc.) of the field study. Individual calibration points are plotted as markers, and the dashed curves represent fit results. With the exception of formic acid fitted using the quadratic model, CIMS Sensitivity S for other acids are fit using the equation $S = a - b\ e^{(-c\ [H2O])}$.

5.0 Signal-to-Noise Ratios

Presented signal-to-noise ratios (SNR) for each measurement were calculated using equation 1:

$$SNR = \frac{E_T t - E_B t}{\sqrt{\sigma_T^2 + \sigma_B^2}} \quad (1)$$

where E_T is the total measurement signal count rate in counts s^{-1}, E_B is the background signal count rate, t is the measurement integration time in seconds, and σ_B and σ_T are the precision (standard deviation) of the background and total (background + analytical signal) signals, respectively. Since $\sigma_T^2 = \sigma_B^2 + \sigma_S^2$, where σ_S is the precision of the analytical signal ($E_S = E_T - E_B$), the denominator can be re-written as $(2\sigma_B^2 + \sigma_S^2)^{0.5}$. If the noise in the background and total signals are purely determined by Poisson statistics (i.e., only by shot noise), then σ_B is equal to $(E_B t)^{0.5}$ and σ_S is equal to $(E_S t)^{0.5}$, and the denominator can be re-written as $(2E_B t + E_S t)^{0.5}$. Finally, since E_S is equal to the product of the mixing ratio [X] and the instrumental sensitivity (or calibration factor) C_f in counts s^{-1} ppt^{-1}, this equation becomes equivalent to Equation 1 from Bertram et al. (2011).[2] For these calculations the unnormalized signals must be used.

Noise for CIMS measurements is often assumed to follow Poisson statistics (i.e., only shot noise) using the equation presented by Bertram et al. (2011).[2] We observe, however, that the noise can be greater than expected. The precision of the various organic acid signals for a representative period are shown in Fig. S5. Sections of stable ambient air, zeros, and standard additions during an automated cycle (see Fig. S3) at approximately 9:50 local time on 15 August 2019 were included for this analysis. The noise (1 standard deviation) in the propanoic, pyruvic, and pentanoic acid signals were found to be nearly equal to the theoretical shot noise (i.e., the square of the signal). The noise in the formic acid data was higher than shot noise by ~20% during a zero and by a factor of ~two during ambient sampling (signal near 90,000 cps). The noise in the fitted acetic acid signal was high by about a factor of two for all observed periods. In addition, we include the nominal mass peak at 187 m/z (i.e., unresolved acetic acid and urea ions) to show that this higher-than-expected acetic acid noise is caused primarily by the ion fitting process. The noise for the total m/z 187 peak was within 15% of the shot-noise value, indicating that the additional noise for acetic acid (high by a factor of ~two) results from the high-resolution peak fitting process – in

particular from the computational separation of the iodide adducts of acetic acid and urea. While several other acids had interfering ions (presented in Fig. S2), the large fitting error likely arises for acetic acid since the urea-adduct differs in mass by only 0.0112 amu (60 ppm) which is much smaller than the differences for the other pairs of overlapping peaks. Ion fitting (i.e., the computational separation of overlapping peak) as a noise source has been modeled.[3] The deviation of precision from shot noise is consistent to the results reported by Yuan et al. (2016) who observed greater than expected noise in several fitted ion signals (ranging up to 2 times greater than shot noise) using a similar instrument and the same fitting software (TofWare v2.5.1) used here.[4] Deviation from theoretical noise may also occur for larger ion signals,[5] consistent with our findings for formic acid.

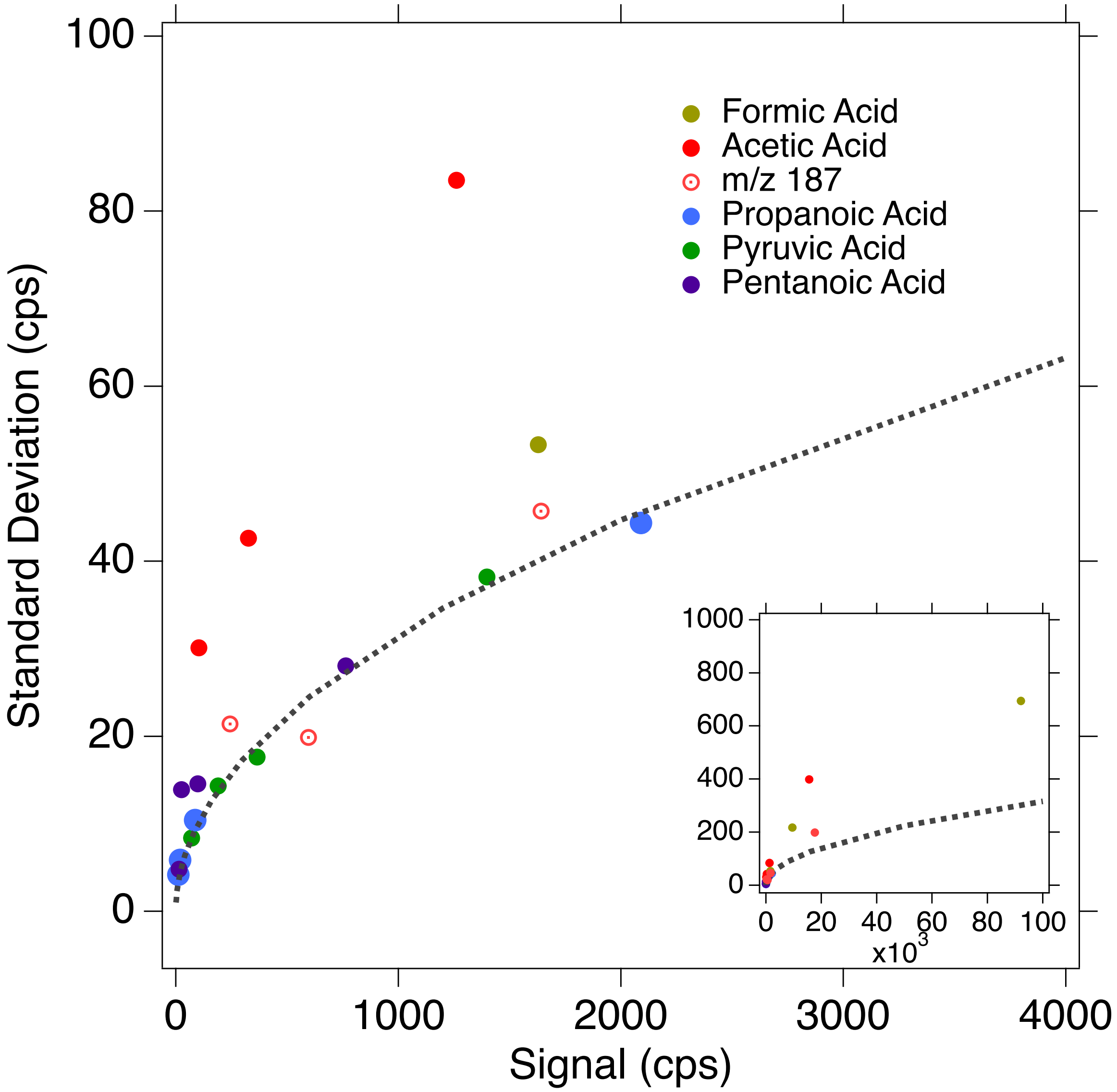

Figure S5 Noise analysis for observed acid signals. Plotted data points include representative zero periods (both cylinder-based and filter-based zeros), ambient air, and standard additions (excluding formic acid). The dashed line represents the theoretical noise (i.e., shot noise) based on Poisson statistics. The inset graph includes expanded axes and shows the addition signal/noise of acetic acid and ambient signal/noise of formic acid.

6.0 Source Apportionment Modeling

The EPA's Positive Matrix Factorization (PMF) Model (Ver. 5.0)[6] was used to investigate source apportionment of organic acids. The parameterization of this model is discussed in the main text (sect. 2.4), and additional information primarily regarding uncertainty inputs is included here. For each of the 10 gas-phase species (i.e., the 5 organic acids of interest, summed NOx, O_3, CO,

NH_3, and HCN), the time-series concentration data was input into the model along with constant uncertainties (by percent of the specified concentration), and detection limits. The model inputs of pyruvic acid data was unique compared to all other compounds since pyruvic acid is presented in the main text with dimensionless units (modified counts) as caused by difficulties in calibration. For this reason, model inputs for pyruvic acid data are also based in dimensionless units. For instances in the dataset with any missing concentration values, we exclude the entire sample.

Ten percent uncertainties were specified for CIMS measured compounds for which standard additions (acetic, propionic, pyruvic, pentanoic) were applied, and a constant 30 % uncertainty was considered for formic acid and HCN (i.e., the CIMS-measured compounds for which there were no standard additions). Constant uncertainties were set as 5 % for the remaining supporting measurements of O_3, NOx, CO, and NH_3. Within the EPA's PMF software, uncertainty may be optionally scaled using a "Weak" certainty setting. This setting was selected only for pentanoic acid data since concentration data were often near the detection limit. The input pentanoic acid uncertainty was increased by a factor of three as a result.

Time series data that falls below specified detection limits results in inflated uncertainty for that species for those particular inputs. Detection limits of organic acids were set to the detection limits specified in the main text in Sect. 2.2.4. The detection limit of HCN was set as 50 pptv, though HCN was rarely observed below 300 pptv. The detection limits for the EPA supporting measurements of O_3, NOx, CO, and NH_3 were set to the EPA reference values.

The output PMF data is presented in the main text using Figure 7 and 8 to show time series data of the output factors and overall contributions, respectively. In addition, we present diel profiles of the source contributions here (Fig. S6).

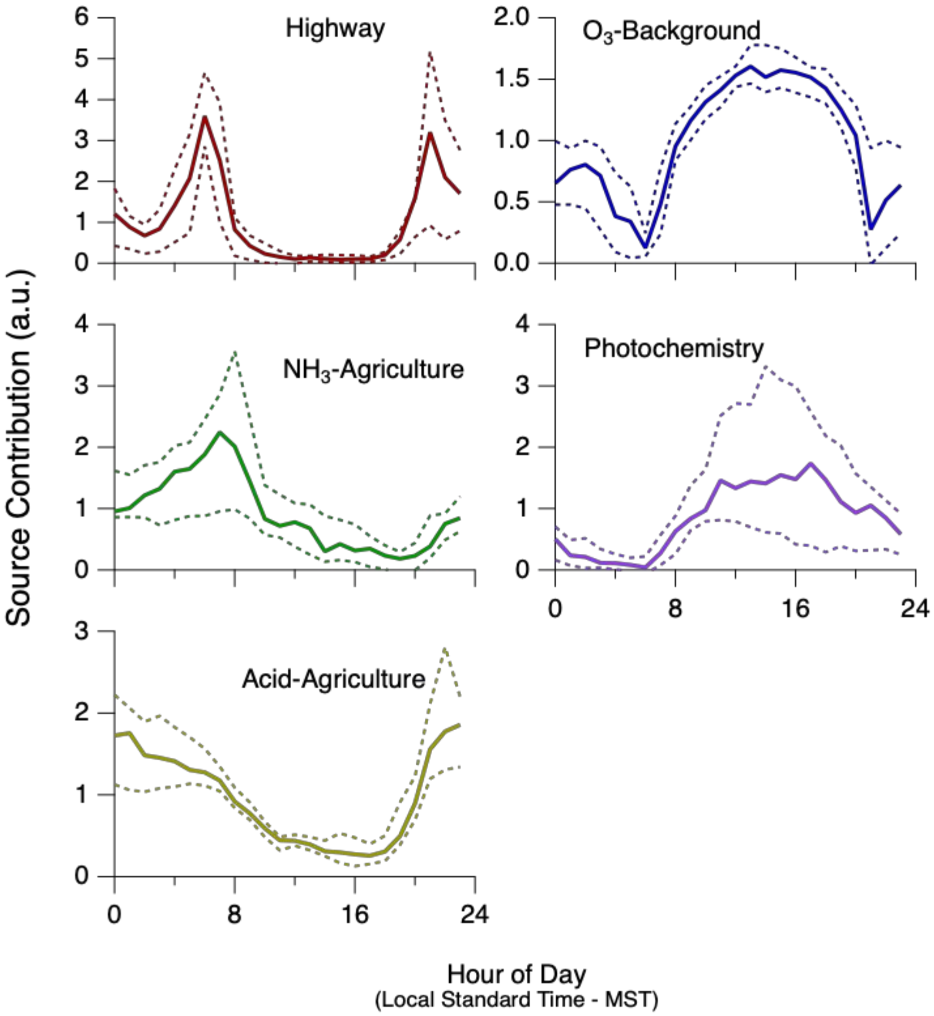

Figure S1 Diel profiles of source contribution for the five factor PMF solution presented in Figure 7 of the main text.

7.0 Highway Influence on Organic Acids

There were several nighttime periods during which the sampled air was greatly affected by emissions from the nearby highway, evident by the wind data (i.e., slow winds from South or Southwest) and enhanced levels of NOx and CO. A few example time periods are shown in Fig. 6 of the main text. In this section we closely inspect examples of these periods. First, we confirm the time alignment between CIMS and supporting measurements. Besides organic acids, nitrous acid (HONO) was also measured by our CIMS (and will be the focus of an upcoming manuscript). We calibrated our CIMS to HONO using a water vapor photolysis method.[7] HONO is known to be

emitted directly from diesel exhaust. For highway influenced periods, the HONO I-CIMS observations were found to increase at the exact same time as the highway tracers CO and NOx (Fig. S7). Here, the start of highway influence occurs with a shift in wind direction towards the south. Increases in NOx, HONO, CO, and CO_2 of approximately 70 ppbv, 0.60 ppbv, 0.40 ppmv, and 20 ppmv were observed, respectively. Organic acid measurements made by the same instrument were not found to have substantial increases until more than 10 minutes later in this example. CO-based scatter plots (Fig. S8) are included to further show the delay in organic acid concentrations. This indicates the presence of a surface source of alkanoic acids in the region.

This observation of delayed organic acid increases during highway influenced periods is representative of the other highway periods. The other significant highway influenced periods resulted from similar shifts in wind along with enhancements in NOx by greater than 40 ppbv, CO by 0.2 ppm, and CO_2 by typically 20 to 30 ppmv. Immediate enhancements in organic acids were minimal for the other similar events. Eventual and substantial increases were always observed several to about 15 minutes after the observed increase in highway tracers.

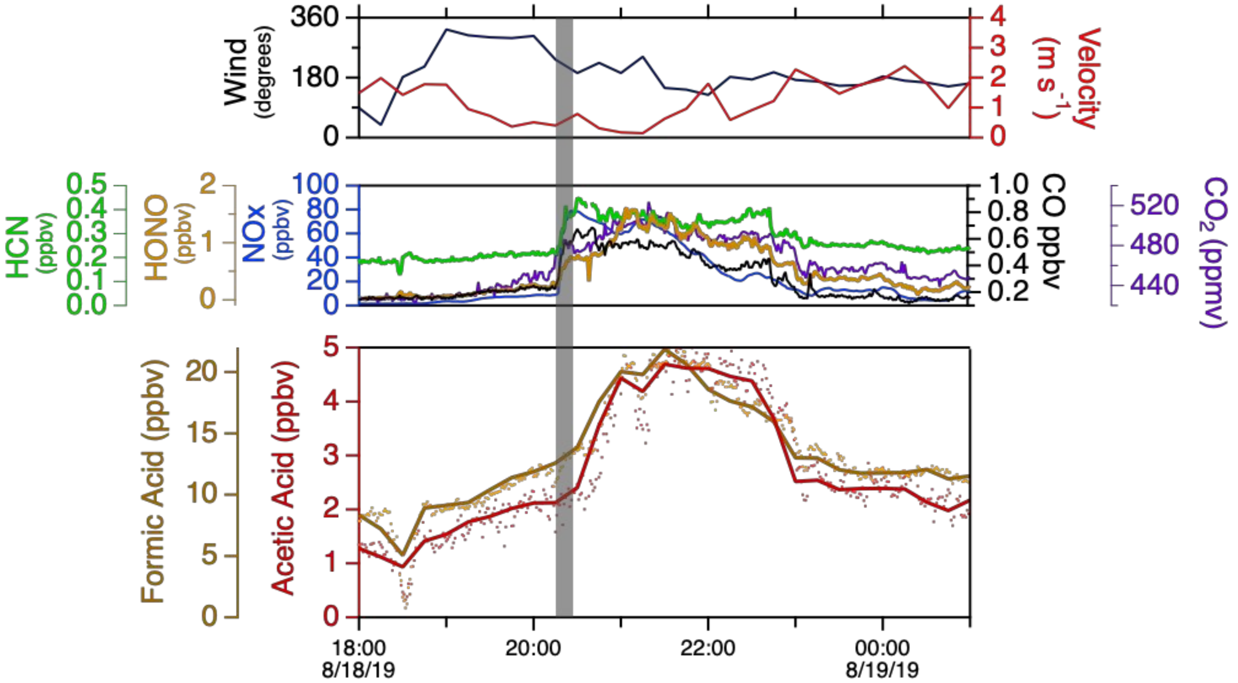

Figure S7 Inspection of highway influenced period on 18 August 2019. The start of the highway period (approximately 20:10 hours local time) represents the start of highway influence. Substantial increases in the organic acids are observed more than 10 minutes after highway influence (after highlighted period) indicating a separate source of acids.

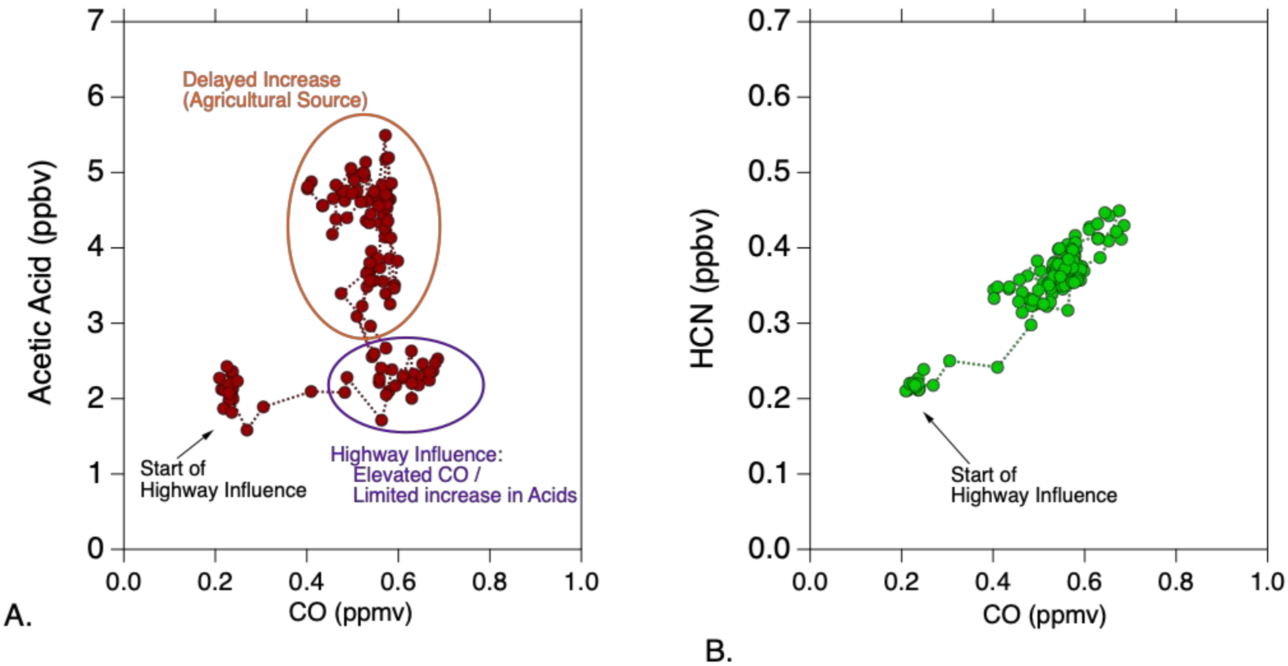

Figure S8 CO-based scatter plots for acetic acid (panel A) and I^- CIMS HCN (panel B) for a 2-hour period of 18 August 2019 beginning at the highway influenced period indicated in Fig. S7.

References:

(1) Lee, B. H.; Lopez-Hilfiker, F. D.; Mohr, C.; Kurtén, T.; Worsnop, D. R.; Thornton, J. A. An iodide-adduct high-resolution time-of-flight chemical-ionization mass spectrometer: Application to atmospheric inorganic and organic compounds. *Environmental science & technology* **2014**, *48* (11), 6309-6317. DOI: https://doi.org/10.1021/es500362a.

(2) Bertram, T.; Kimmel, J.; Crisp, T.; Ryder, O.; Yatavelli, R.; Thornton, J.; Cubison, M.; Gonin, M.; Worsnop, D. A field-deployable, chemical ionization time-of-flight mass spectrometer. *Atmospheric Measurement Techniques* **2011**, *4* (7), 1471.

(3) Cubison, M.; Jimenez, J. Statistical precision of the intensities retrieved from constrained fitting of overlapping peaks in high-resolution mass spectra. *Atmospheric Measurement Techniques* **2015**, *8* (6), 2333-2345.

(4) Yuan, B.; Koss, A.; Warneke, C.; Gilman, J. B.; Lerner, B. M.; Stark, H.; de Gouw, J. A. A high-resolution time-of-flight chemical ionization mass spectrometer utilizing hydronium ions (H 3 O+ ToF-CIMS) for measurements of volatile organic compounds in the atmosphere. *Atmospheric Measurement Techniques* **2016**, *9* (6), 2735-2752.

(5) C. Corbin, J.; Othman, A.; D. Allan, J.; R. Worsnop, D.; D. Haskins, J.; Sierau, B.; Lohmann, U.; A. Mensah, A. Peak-fitting and integration imprecision in the Aerodyne aerosol mass spectrometer: effects of mass accuracy on location-constrained fits. *Atmospheric Measurement Techniques* **2015**, *8* (11), 4615-4636.
(6) EPA. Positive Matrix Factorization (PMF) 5.0 Fundamentals & User Guide. Report No. EPA-600/R-14/108. Office of Research and Develpment, Washington, DC, USA. **2014**.
(7) Lindsay, A. J.; Wood, E. C. Comparison of two photolytic calibration methods for nitrous acid. *Atmospheric Measurement Techniques* **2022**, *15* (18), 5455-5464. DOI: https://doi.org/10.5194/amt-15-5455-2022.
